# Supplementary material for: High frequency head impact exposure changes hippocampal sharp-wave ripple architecture
Source: PLoS One. 2026 Jan 9;21(1):e0336990. doi: 10.1371/journal.pone.0336990 (PMC12788646; doi:10.1371/journal.pone.0336990)
Supplement: Attachment — Submitted filename: Reviewer Response_FINAL.pdf [file pone.0336990.s002.pdf]

High frequency head impact exposure changes hippocampal sharp-wave ripple architecture

We thank the reviewers for their critiques and kind words regarding the potential impact of this research. We believe that the manuscript is much improved with these recommendations. We address each of the comments below in detail, and in the manuscript with track changes.

Reviewer #1:

Summary

This manuscript explores the relationship between head injury and hippocampal dynamics, with in vitro measurements in mice. This manuscript's main question: how do repeated head impact change oscillatory patterns in the hippocampus CA1 region, and more precisely how repeated impacts affect sharp waves and ripples? The mode of injury is described as high-frequency head impact (HFHI), which has low impact force, but presents the effect of multiple cumulative small impacts, such as in football/soccer, or other action sports. The authors have published using this HFHI method previously. After the experimental manipulation, the authors record field potential activity in the hippocampus CA1 and compare the changes in the spike-wave and ripple combo activity, along with the activity of the fast ripples, and gamma periods. Across these measurements, it appears like HFHI mice show lower power of activity across these frequency boundaries. Thus, overall, the authors propose an interesting study, with results that help describe fast oscillatory components that change with injury. While speaking more as a generalist of field potentials, the methods appear well executed, and in general the data acquisition and analysis also seem to be well done, though I have a few questions. Overall, the discussion and conclusions appear sound. This paper has the potential to solidly advance our knowledge in the neurophysiological changes emanating from HFHI.

Major points:

Results

1. Line 184: it would help to see certain examples of data reduction - so how the data gets to be reduced to one data point per animal. A figure (a histogram, for example) showing how the data from multiple recording periods gets to be reduced to one value. This would also provide context as to the variability of measurements per animal.

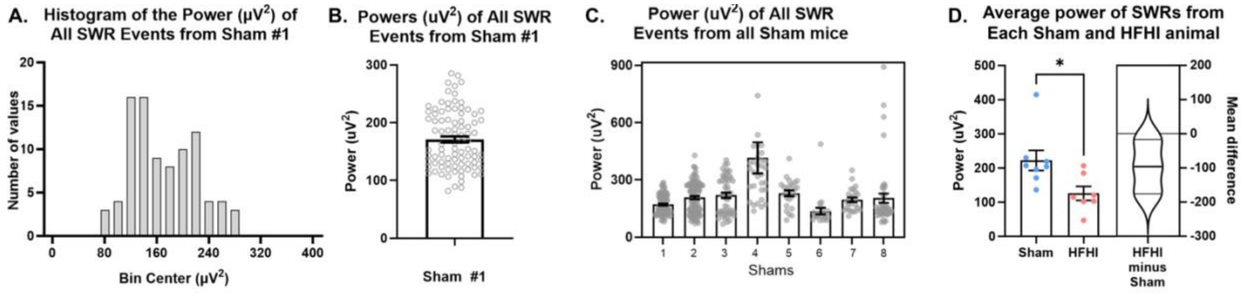

Above is an example of the data reduction. For the example, we show the SWR Power data from Sham #1. The recording is quantified, giving individual power values for each SWR. Those are averaged to provide a singular value for of SWR power for each animal. (A) Histogram of all the SWR events recorded for Sham #1. (B) Mean and standard error of the powers of all SWR events from Sham #1. (C) Mean and standard error of the powers of all SWR events for all the Sham animals in order, demonstrating similar distributions of data across animals. (D) Mean values of powers of SWR events for all animals; the same graph as included in the manuscript.

2. Between lines 197 and 202: what was it, visual inspection or automated detection? While I'm confident the authors used a reliable algorithmic approach to the detections, the explanations "flip" a few times between the "visual inspection" and "automatic detection". This deters from the comprehension.

We apologize for the confusion. We used visual inspection to confirm the presence or absence of SWR in our slices (Data in Fig 1B) after electrode placement and prior to recording. However, after collecting the recordings, we used an automated detection software to the analyze average amplitude, frequency, duration and power of SWR events from gap-free recordings. We have clarified this in the opening paragraph of the results.

This has been updated in Line 169 and line 206.

3. Figure 1D: what is the meaning of the zoomed-in spectrogram? Unless this carries specific information – this can be removed.

The spectrograms have been removed.

4. Line 220: the SWR power should be defined more clearly - is it the spike wave amplitude, or is it the area under the "SWR curve"? This explanation would provide more clarity as to the power being measured.

This has been clarified in the text (this is now Line 225): "…total SWR power (area under the SWR curve)…"

5. Line 232 and figure 3: it appears that the ripples are 10-12 cycles long… this seems very long compared with the examples in Figure 1 which are 5-6 cycles. This should be established – maybe with a sample trace next to the figure?

The examples in Figure 1 have been replaced with more representative traces; the number of ripples in the traces are n=14 ripples for Sham and n=10 ripples for HFHI.

6. Similar to #4, but here likely more straightforward… Figure 3C presents ripple power is it the area under the spectrogram curve between 120 Hz and 220 Hz?

This has been clarified in the text at line 242: "Ripple power (area under the curve)…"

7. Line 245: the authors have explained the selection of their gamma frequency band between 20 Hz and 50 Hz: while literature is not always fixed on the frequency, some examples give low gamma between 30-70 Hz and high gamma between 70-150 Hz (e.g., Catanese et al., 2016, JNeurophysiol). Without advocating for change, at least the authors should explain their choice.

We agree there is misalignment amongst the wider body of literature when it comes to the identification of low gamma in regards to SWR filtering. A general search of low gamma in SWR will reveal the widespread use of 20-50Hz for filtering low-gamma (PMID: 36572725; 22920260; 32429703, 20200521), and we have now referenced two of these papers in our manuscript. Other groups use 25Hz (PMID: 30121451) or 30Hz (PMID: 26135716).

The following is now updated in Lines 253 of the main text, with relevant references: "…and low-gamma bandwidths [22, 29]."

<u>Minor points:</u>

Abstract
• Line 51: what does it mean in the absence of pathology? Anatomical disturbance?

This line has been updated: "…and causes no cell death, no axonal damage, no tau or amyloid accumulation, and no inflammation, yet results in impaired cognitive function…"

Introduction
• line 68: it would help if examples of observable pathology were provided (lesions, cortical thinning?)

Further clarification was added to this line (now Line 69) in the text: "The observable pathology characteristics of CTE of axonal injury, inflammation, and tau accumulation does not directly correlate to symptoms of repeat head impacts…"

Methods

• line 160: The authors should provide more information concerning the selection of the depth - was this to capture the best LFPs?

Further clarification was added to this line in the text: "This depth enabled optimal SWR detection without slice damage, and a standardized depth reduced variability across slices as variability in slice depth can affect SWR detection."

• line 166: please clarify the order of the first two sentences in "Data analysis" - it might be better to flip them?

Thank you – these sentences have been swapped.

Results

• Line 200: is it really an artifact? More simple word – a cause?

This has been changed to "cause" in the main text (now Line 211).

• Line 215 and figure 2B: the terminology is equivocal - is it frequency or rate of SWR events? Rate seems better – it feels like frequency could be confusing as it is used to describe the oscillatory phenomena.

Thank you – this has been changed to "rate" throughout the text and in the figures.

• Lines 220 to 228: While this might be a specific personal style, there is usually no need to provide description and citations here… this should be in the introduction or discussion; same goes for lines 243-246… this should be checked re: the journal's style.

We have removed these lines from the results and into the introduction.

• Figure 4D: The Y axis should not have "gamma ripple" - this seems like a mistake.

The axis has been changed.

• Line 336: CCI should be defined.

We have replaced CCI with "severe experimental brain injury" (now Line 338).

<u>Reviewer #2:</u>

In their manuscript, Chapman et al. investigate the impact of high-frequency head impacts (HFHI) on hippocampal function in mice, focusing on sharp-wave ripples (SWR), a known biomarker of memory and cognition. The authors demonstrate that although SWRs remain structurally intact post-HFHI, their amplitude, power, and ripple cycle count are significantly reduced compared to controls. These findings suggest that HFHI impairs intrinsic hippocampal network activity, potentially contributing to cognitive deficits observed in the absence of overt brain pathology. The work provides interesting insight into subtle neurophysiological changes following repetitive head trauma
I have a few comments and suggestions regarding the work listed as they appear throughout the manuscript.

We thank the reviewer for their concise summary of our paper and thoughtful comments. We hope to address all the comments below and improve upon the existing paper prior to publication. Our responses to the comments are in blue font on this page and are made in track changes in the body of the paper.

- Abstract:

The notion of 'reduced architecture' in the last sentence of the abstract feels imprecise. I suggest the authors use a different terminology

This has been changed to: "HFHI alters hippocampal SWR architecture, reducing metrics including SWR amplitude and power, which may play a role in impaired cognition…"

- Introduction:

The last sentence of the introduction (lines 105-106) should include that the work was performed on acute brain slices

This has been changed: "…hippocampal SWR in acute brain slices." This is now Line 110.

- Methods:

Electrophysiology
Lines 161-163, It's not totally clear how many 2-min-long recordings were performed on each slice. It could be useful to also provide the average number of slices recorded per animal and the average number of recordings performed on each slice.

The data for each mouse was obtained from a single 120-second recording taken from the central-most hippocampal slice per animal after slice acclimatization. The experimenter binarily classified all recordings as having SWR or not if the experimenter could visually detect them at least 10 minutes after electrode placement in gap-free recordings. If the experimenter could detect SWR within this time period, the electrode remained in the slice for a minimum of 30 minutes to allow the slice to acclimate to the recording conditions. A 2-minute-long recording from each slice was recorded in current clamp. Only a singular recording from each slice was used to generate data. In 3 mice we detected SWR from two slices per mouse, and in these cases we averaged the final datapoints for each metric so that we only have a singular datapoint per mouse.

The data recorded from each sham slice is presented above in response to a comment from Reviewer 1.

In line 171, the authors mention the 'best 2-minute recording'. What are the actual criteria used to sort or classify recording quality?

With a single animal, the quality of the SWR dropped off over the 30-minute rest period - so we did not use that recording. Instead, we moved the electrode, allowed it to remain in the slice for 30 minutes, and we recorded a new 2-minute recording that resulted in clear SWR with a good signal to noise ratio. We used this recording for our analysis from this mouse. We have removed this phrase from the manuscript as it is not a good descriptor.

Data Analysis
The authors should define in a few words the outputs from the Matlab software they quantified

The following has been added to the manuscript: "Sharp-wave and ripple events were automatically detected using open-source MATLAB software which enables quantification of metrics including, but not limited to, rates, amplitudes, and durations of events in raw or filtered field potentials [22]."

Statistics
Prior to the use of parametric tests, did the authors check for normality and homoscedasticity? In case if violation, did the authors perform corrections?

Clarification regarding normality checks has been added to the statistics section: "Prior to statistical analysis, all data was checked for normality using the Shapiro-Wilk test. No data violations were found."

- Results:
In line 198, because the authors removed one outlier animal in the HFHI group, shouldn't it be 7 out of 10 for the HFHI group?

The figure is showing how many acute mouse brain slices that we could detect SWR events in. We analyzed 11 animals per group, with sharp wave ripples visually detected in 8 of the 11 animals in both the sham and HFHI.

As discussed in the statics section of the methods, after analyzing the resulting sharp wave ripple data, we found that multiple SWR metrics of one of HFHI animals violated outlier testing. That animal was therefore removed from all analyses. Thus, our final group sizes were n=8 Sham and n=7 HFHI.

We have now also clarified this in the results with the following statement: "In later statistical testing of the data, one HFHI brain failed outlier testing, leaving a final n=8 Sham and n=7 HFHI." This is now Lines 209.

The panel A in Figure 1 may be slightly revised to make it more obvious that the head impacts protocol occurred during 6 consecutive days, for example by showing the 6 different points corresponding to each of the 6 days.

The panel has been revised to show the six consecutive days of repeat mild head impacts received during the experimental time course.

In Figure 1, panels D and F, the Y axis for the spectrograms are missing.

The spectrograms have been removed per Reviewer 1's comments.

As mentioned above regarding the methods, the features extracted from SWR events should be more clearly explained. For example, in lines 216-217, about the quantification of SWR duration, the authors refer to the text in the methods section, but there is no detailed information in this section. This additional information should be provided directly in the manuscript. Some schematic panels illustrating the extracted features may be also added in Figures 2-4

Thank you for this feedback – on top of our previous comments we have added the following in the methods:

- *Overview of metrics software analysis*
  - Sharp-wave and ripple events were automatically detected using open-source MATLAB software which enables quantification of metrics including, but not limited to, rates, amplitudes, and durations of events in raw or filtered field potentials. This is now Lines 168.
- *Additional information regarding how the swrAnalysis program works*
  - As previously described, this software detects SWR events by flagging all instances of lasting at least 10 milliseconds that are concurrently elevated greater than four standard deviations above baseline in both the sharp wave (1-30 Hz) and ripple (120-220 Hz) frequencies [22]. After events after detected, events are analyzed for metrics such as duration (start and end time), power (area under the curve at the analyzed frequency), amplitude (baseline subtracted from sharp wave peak), and number of cycles (filtered only, eg ripple cycles) from start to end of SWR event [22]. This is now on Lines 171.

Some of the results reported by the authors fall very short on reaching the threshold for significance while displaying large Cohen effect sizes (eg SWR duration, gamma power, fast ripple power). Thus, the authors should consider increasing the sampling size to strengthen their results.

With an ongoing robust discussion of statistics occurring in science, we wanted to present our data as a combination of estimation and frequentist statistics. In our study, we are sufficiently powered to assess our

primary outcome measures – which is SWR rate and amplitude. However, we also wanted to represent different ways of analyzing secondary measures that have higher levels of variability.

But we do understand the concern. As a compromise, we have removed the Cohens d plots from all figures where the data did not pass the threshold of significance in our frequentist tests.

Line 326, I may be mistaken, but it looks like 'CCI' was not defined before.
We have replaced CCI in this line (now line 320) and in multiple other places in the text with the phrase "severe experimental brain injury".

Lines 351-353, The authors should more explicitly state that this statement is a hypothesis. Line 354, As mentioned above, this lack of statistical significance may result from a low sampling.

We have changed the paragraph to read (now line 350):

While we saw trends that suggest a broadband decrease in hippocampal power, this did not reach statistical significance so this remains a hypothesis with the current sample size. We did however, observe a decrease in fast ripple cycles in HFHI mice.
